# Performance Enhancement in N_2_ Plasma Modified AlGaN/AlN/GaN MOS-HEMT Using HfAlO_X_ Gate Dielectric with Γ-Shaped Gate Engineering

**DOI:** 10.3390/ma14061534

**Published:** 2021-03-21

**Authors:** Shun-Kai Yang, Soumen Mazumder, Zhan-Gao Wu, Yeong-Her Wang

**Affiliations:** Department of Electrical Engineering, Institute of Microelectronics, National Cheng Kung University, Tainan 701, Taiwan; kyle30501@gmail.com (S.-K.Y.); soumen.mazumder9@gmail.com (S.M.); andy85cc@gmail.com (Z.-G.W.)

**Keywords:** AlGaN/AlN/GaN, MOS-HEMT, HfAlO_X_, HfO_2_, interface trap density, post-deposition annealing (PDA), Γ-shaped gate; flicker noise

## Abstract

In this paper, we have demonstrated the optimized device performance in the Γ-shaped gate AlGaN/AlN/GaN metal oxide semiconductor high electron mobility transistor (MOS-HEMT) by incorporating aluminum into atomic layer deposited (ALD) HfO_2_ and comparing it with the commonly used HfO_2_ gate dielectric with the N_2_ surface plasma treatment. The inclusion of Al in the HfO_2_ increased the crystalline temperature (~1000 °C) of hafnium aluminate (HfAlO_X_) and kept the material in the amorphous stage even at very high annealing temperature (>800 °C), which subsequently improved the device performance. The gate leakage current (I_G_) was significantly reduced with the increasing post deposition annealing (PDA) temperature from 300 to 600 °C in HfAlO_X_-based MOS-HEMT, compared to the HfO_2_-based device. In comparison with HfO_2_ gate dielectric, the interface state density (D_it_) can be reduced significantly using HfAlO_X_ due to the effective passivation of the dangling bond. The greater band offset of the HfAlO_X_ than HfO_2_ reduces the tunneling current through the gate dielectric at room temperature (RT), which resulted in the lower I_G_ in Γ-gate HfAlO_X_ MOS-HEMT. Moreover, I_G_ was reduced more than one order of magnitude in HfAlO_X_ MOS-HEMT by the N_2_ surface plasma treatment, due to reduction of N_2_ vacancies which were created by ICP dry etching. The N_2_ plasma treated Γ-shaped gate HfAlO_X_-based MOS-HEMT exhibited a decent performance with I_DMAX_ of 870 mA/mm, G_MMAX_ of 118 mS/mm, threshold voltage (V_TH_) of −3.55 V, higher I_ON_/I_OFF_ ratio of approximately 1.8 × 10^9^, subthreshold slope (SS) of 90 mV/dec, and a high V_BR_ of 195 V with reduced gate leakage current of 1.3 × 10^−10^ A/mm.

## 1. Introduction

In recent years, the wide band gap semiconductors have been widely used in high power electronic applications due to its high power density and high power conversion efficiency [1]. As of today, the AlGaN/GaN based high electron mobility transistors (HEMTs) are the most promising devices for high power and high frequency applications due to their unique properties such as wide band gap (3.4 eV of GaN), large breakdown field (>3 MV/cm), high density of two-dimensional electron gas (2 DEG) (~10^13^/cm^2^), low intrinsic carrier density, and high saturation velocity (~$2\times 10^{7}/{\mathrm{cm}}^{2}$) [2,3,4,5]. Due to the safety and power saving concerns, the recessed gate GaN-based HEMTs are desirable to realize the normally-off operation [6]. However, Ki-Sik et al. and Zhe at al. reported that the electron transport characteristics could be seriously affected by the interface states in fully recessed HEMTs, due to the completely etched AlGaN barrier layer [7,8]. 

In order to avoid the degradation of the transport characteristics of the carrier the partially recessed GaN HEMT structure was proposed [9]. The Cl_2_-based inductive coupled plasma (ICP) dry etching has been widely used, to realize the partially recessed-gate structure. However, the gate recess process induced the trap states in the device, leading to the severe gate leakage, worse current collapse, and very low breakdown voltage (V_BR_) [6,10]. Baik et al. reported that the N_2_ plasma treatment could effectively improve the surface morphology and ohmic contacts in GaN HEMTs by reducing the N_2_ vacancies, created by ICP dry etching [11].

In addition to the N_2_ surface treatment, to reduce the gate leakage current significantly, the metal oxide semiconductor high electron mobility transistor (MOS-HEMT) with an insulating dielectric have also been widely investigated. Numerous gate dielectrics have been experimented, such as SiO_2_ [12], AlN [13], Al_2_O_3_ [14], MgCaO [15], HfO_2_ [16], ZrO_2_ [17], TiO_2_ [18], etc. The dielectric layer not only can suppress the gate leakage current, but also can be used as a passivation layer to suppress the current collapse phenomenon [19]. Among the many used dielectrics, HfO_2_-based MOS-HEMTs can achieve much more efficient electrostatic control due to their high dielectric constant (k). However, due to the insufficient barrier height, the HfO_2_-based GaN MOS-HEMT suffers from a high gate leakage current, which subsequently deteriorates the device performance [20]. Thus, to reduce the gate leakage current without the reduction of gate controllability, the HfO_2_-based stack gate dielectric layer such as HfO_2_/Y_2_O_3_ [21], HfO_2_/Al_2_O_3_ [16], HfO_2_/AlN [22] or Hf-ternary oxide HfSiO_X_ [20], HfZrO [23], etc. have also been investigated. However, owing to the lower crystalline temperature, e.g., Y_2_O_3_ (~ <425 °C) [24] and HfZrO (500~550 °C) [25], the respective MOS-HEMT might not be applicable in a high temperature.

A previous study has shown that the inclusion of Al into HfO_2_ could improve the interface properties and reduce the gate leakage current in hafnium aluminate (HfAlO_X_)-based MOS-HEMTs [26,27]. HfO_2_ provides a high dielectric constant (k ~ 25) but poor conduction band offset to GaN (~1.51 eV), while Al_2_O_3_ offers a larger conduction band offset to GaN (~1.96 eV) but it has a relatively lower dielectric constant (k ~ 9) [27]. To improve the channel controllability a high-k material is preferred, whereas a large conduction band offset is required to reduce the gate leakage current.

In addition, a high temperature sustainability is much more important for a high power application. Since the phase change from amorphous to crystalline will rise to the leakage current, the crystalline temperature of HfO_2_ (~400 °C) is much lower than Al_2_O_3_ (~900 °C) [27]. Previous reports also suggested that the HfO_2_ suffers severely from the interface state and border state densities than Al_2_O_3_, which affects the device performances significantly [26,28]. Thus, by incorporation of Al into HfO_2_ the thermal stability of the ternary compound, i.e., HfAlO_X_ could be improved significantly. 

The crystallization temperature of HfAlO_X_ could give rise to ~1000 °C with 45.5% of Al [26]. The conduction band offset of HfAlO_X_ is also much higher (~1.61 eV) [27], with a high dielectric constant (k~14) [29]. Moreover, due to the incorporation of Al, the interface state density is effectively reduced with the passivation of the dangling bond in HfAlO_X_, which helps the improvement in the device performance significantly [30]. To date, the direct observation of the enhancement of device performance in HfAlO_X_-based MOS-HEMTs with N_2_ surface plasma alteration using the Γ- shaped gate structure, has not yet been investigated. 

With this aim in mind, in this work, we have demonstrated the improvement of device performance in Γ- gate AlGaN/AlN/GaN MOS-HEMT with the Al doped HfO_2_ gate dielectric and N_2_ surface plasma modulation. The inclusion of Al into HfO_2_ increased the crystalline temperature of HfAlO_X_, which reduced the gate leakage current at a high PDA temperature (~600 °C). The interface state density of HfAlO_X_ MOS-HEMT is reduced nearly one order of magnitude, which improved the hysteresis behaviour of the device. Moreover, the use of gate field plate (FP) increased the breakdown voltage (V_BR_ ) of the device. The N_2_ plasma treated Γ-shaped gate HfAlO_X_-based MOS-HEMT exhibited a decent performance with I_DMAX_ of 870 mA/mm, G_MMAX_ of 118 mS/mm, threshold voltage (V_TH_) of −3.55 V, higher I_ON_/I_OFF_ ratio of approximately 1.8 $\times 10^{9}$, subthreshold slope (SS) of 90 mV/dec, and a V_BR_ of 195 V with the reduced gate leakage current of $1.3\times 10^{-10}$ A/mm. 

## 2. Materials and Methods

The AlGaN/AlN/GaN-epitaxy was grown on a 6-inch low resistive (111) Si substrate by the metal organic chemical vapour deposition (MOCVD) system. The epitaxial layer consists of a 5.5 μm GaN buffer layer, a 200 nm GaN channel layer, a 1 nm AlN layer and a 25 nm Al_0.23_Ga_0.77_N barrier layer, and a 2 nm GaN cap layer. The measured Hall mobility, sheet carrier concentration, and sheet resistance were found to be 1800 cm^2^/V·s, 8 × 10^12^ cm^−2^, and 434 Ω/◻, respectively.

**Figure 1:**
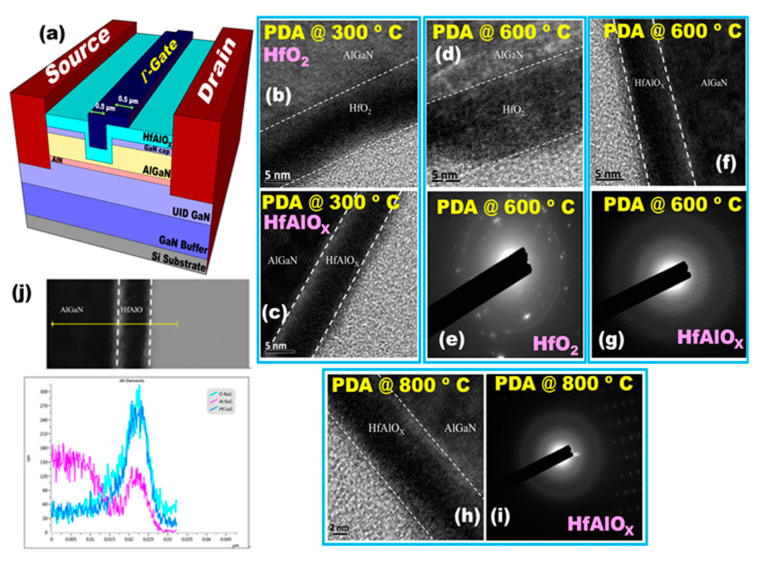
(**a**) Schematic illustration of Γ-shaped gate metal oxide semiconductor high electron mobility transistor (MOS-HEMT). TEM image of Γ-gate (**b**) HfO_2_ and (**c**) HfAlO_X_ MOS-HEMT with the post deposition annealing (PDA) at 300 °C. (**d**) TEM image and (**e**) the selected area diffraction pattern (SADP) of Γ-gate HfO_2_ MOS-HEMT and (**f**) TEM image and (**g**) selected area diffraction pattern (SADP) of Γ-gate HfAlO_X_ MOS-HEMT with PDA at 600 °C. (**h**) TEM image and (**i**) SADP of Γ-gate HfAlO_X_ MOS-HEMT with PDA at 800 °C. (**j**) EDX line scan of HfAlO_X_.

To achieve the device isolation the mesa etching was employed by using the inductive coupled plasma reactive ion etching (ICP-RIE) system under the Cl_2_/BCl_3_ environment. After that, the source and drain regions were defined using UV-photolithography, followed by Ti/Al/Ni/Au (25/150/30/120 nm) metal stacks were deposited by the electron beam (e-beam) evaporation system. Then, the rapid thermal annealing (RTA) took place at 875 °C for 45 s in N_2_ ambient to ensure the good ohmic contact. After that, the gate recess area was defined by the ELS-7500 EX electron beam lithography (EBL) system. Subsequently, the GaN and AlGaN layers under the gate were partially recessed while using low power ICP-RIE. After that, the N_2_ plasma treatment was done by RF-sputter and the sample was transferred to the atomic layer deposition (ALD) chamber (Picosun) to deposit the gate dielectric. The 10 nm thick hafnium aluminate (HfAlO_X_) was deposited as the gate dielectric by ALD at 250 °C. As for the 10 nm HfAlO_X_ deposition, three cycles of Al_2_O_3_ were first deposited, then four cycles of HfO_2_, and one cycle of Al_2_O_3_ were cyclically deposited until the total thickness of 10 nm. Then, the sample was coated with an electron resist and baked at 180 °C for 7.5 min. After that, the Γ-shaped gate region was defined by the e-beam lithography system. Finally, the Ni/Au (80/100 nm) gate metal stack was deposited by the e-gun evaporator. In addition, (i) the Γ-shaped gate MOS-HEMT with the HfO_2_ gate dielectric, (ii)  Γ-shaped gate HEMT, and (iii) non-recessed HEMT (C-HEMT) were also fabricated following the same process as the control samples. In addition, to examine the high temperature sustainability of HfAlO_X_, the MOS-HEMTs were fabricated with three different post deposition annealing (PDA) temperatures, i.e., 300, 600, and 800 °C for 1 min, and compared with HfO_2_ MOS-HEMT. The schematic of the AlGaN/AlN/GaN Γ-shaped gate MOS-HEMT is shown in Figure 1a. All the devices were fabricated with the same gate length (L_G_ = 0.5 µm) and L_GD_/L_SD_ (2/2 µm). 

## 3. Results and Discussion 

Figure 1b–i shows the typical transmission electron microscope (TEM, JEM-2010 Electron Microscope; JEOL Co. 200 KV) images of the HfO_2_- and HfAlO_X_-based MOS-HEMT. Without the intermixing of layers, a quite smooth oxide/GaN interface was observed. In order to compare the thermal stability of HfO_2_ and HfAlO_X_, the PDA was done at 300 °C and 600 °C for 1 min and the characteristics were analyzed. Figure 1b,c shows the TEM images of the Γ-gate HfO_2_ and HfAlO_X_ MOS-HEMTs with the PDA at 300 °C. It was noticed that the gate dielectrics were in amorphous stage, i.e., no crystalline lattice fringes were found after the PDA at 300 °C. From Figure 1d, it can be observed that the amorphous phase of HfO_2_ was changed to polycrystalline with the increasing annealing temperature from 300 to 600 °C. In addition, the selected area diffraction pattern (SADP) was used to analyze the details of the crystal diffraction by TEM, as shown in Figure 1e. Some diffraction peaks were found in the 600 °C annealed HfO_2_ MOS-HEMT. As the AlGaN is a single crystal, thus, the diffraction ring is about the HfO_2_ crystallization. 

On the other hand, from Figure 1f,g it can be clearly understood that the HfAlO_X_ was still in the amorphous stage at 600 °C. No diffraction peak was found in the SADP image, which further proved the aforementioned statement. In order to further analyze the thermal stability of HfAlO_X_, the material was annealed at 800 °C for 1 min. The TEM and SADF images proved that HfAlO_X_ was still amorphous, as shown in Figure 1h,i. The results revealed that the crystallization temperature of HfO_2_ was improved to a great extent by the incorporation of Al. Figure 1j shows the energy-dispersive X-ray spectroscopy (EDX) measurement with a line scan mode of HfAlO_X_ film. It can be observed that the Hf and Al atoms are distributed uniformly. The ratio between Al and Hf in HfAlO_X_ is about 1:2.

**Figure 2:**
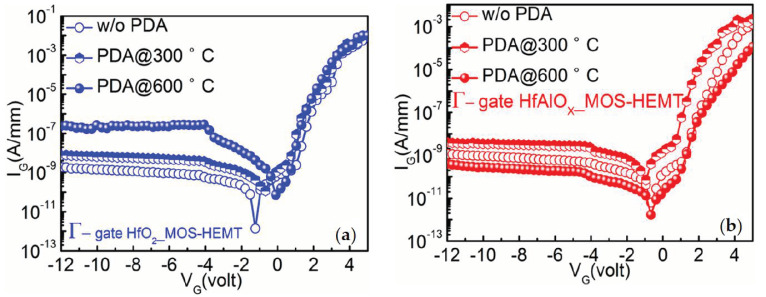
Comparison of gate leakage characteristics of (**a**) Γ-gate HfO_2_ MOS-HEMT and (**b**) Γ-gate HfAlO_X_ MOS-HEMT with the PDA at 300 and 600 °C and without the PDA.

Figure 2a,b shows the gate leakage (I_G_) characteristics of HfO_2_- and HfAlO_X_-based MOS-HEMTs with different PDA temperatures. The gate leakage current of HfO_2_ MOS-HEMT was increased approximately two orders of magnitude with the increasing annealing temperature to 600 °C, as shown in Figure 2a. As the TEM images confirmed that the HfO_2_ phase changes from amorphous to polycrystalline with the increasing PDA temperature, thus the additional high leakage path along the grain boundaries through the poly crystalline dielectrics increased I_G_ with the PDA temperature [26]. The I_G_ values (@ V_G_ = −12 V) were found to be approximately 10^−7^ and 10^−8^ A/mm (10^−9^ A/mm) orders of magnitude for Γ-gate HfO_2_ MOS-HEMTs with 600 and 300 °C PDA (w/o PDA). From Figure 2b, it can be observed that the I_G_ values were decreased about two orders of magnitude with the increasing annealing temperature from 300 to 600 °C in HfAlO_X_ MOS-HEMTs. The decreased trap density with the increasing annealing temperature might be one possible reason for the reduction in the leakage current [26]. The I_G_ (@ V_G_ = −12 V) was increased from 10^−9^ to 10^−8^ mA/mm, when the temperature raised from room temperature (RT) to 300 °C, and then the gate leakage was reduced approximately to 10^−10^ mA/mm, with further increments of the PDA temperature to 600 °C. At a higher temperature, the growth of SiO_2_ at the interface resulted in the reduction in I_G_ [26].

**Figure 3:**
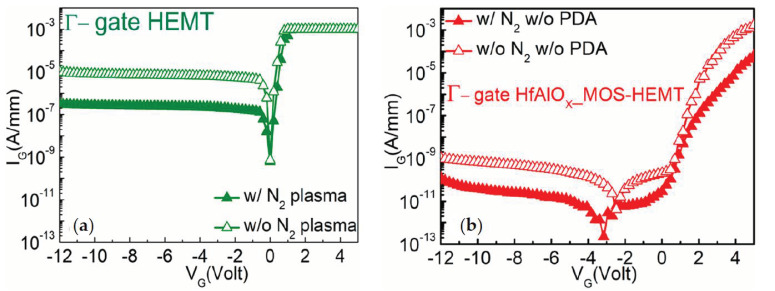
Comparison of gate leakage characteristics of (**a**) Γ-gate HEMT and (**b**) Γ-gate HfAlO_X_ MOS-HEMT with and without the N_2_ surface plasma treatment.

In order to analyze the effects of nitrogen (N_2_) plasma treatment after ICP etching, on the gate leakage current, the I_G_–V_G_ characteristics were calibrated from Γ-gate HEMT and HfAlO_X_ MOS-HEMTs with and without the N_2_ plasma treatment, as shown in Figure 3a,b. It can be clearly observed that the gate leakage current was reduced more than one order of magnitude in Γ-gate HfAlO_X_ MOS-HEMTs, whereas the I_G_ was reduced approximately two orders of magnitude in the Γ-gate partially recessed HEMT by the N_2_ plasma treatment. The N_2_ vacancies, created by ICP dry etching, could be filled by the nitrogen radicals generated from the pure N_2_ plasma. In addition, reducing the dangling bonds by forming Ga-N bonds resulted in activating the surface, which correspondingly reduced the gate leakage current [31].

**Figure 4:**
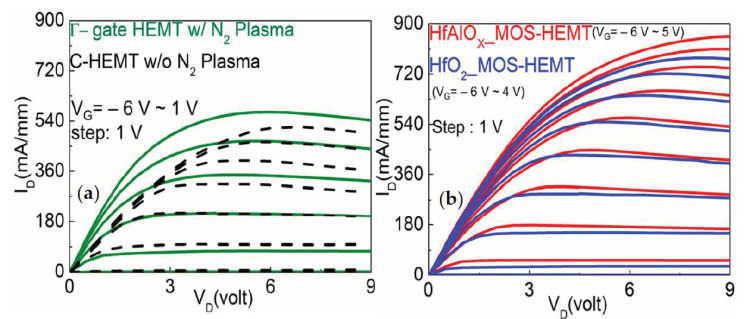
Comparison of drain current-voltage (I_D_–V_D_) characteristics of (**a**) Γ-gate HEMT (with the N_2_ plasma treatment) and non-recessed HEMT (w/o the N_2_ plasma treatment) and (**b**) Γ-gate HfO_2_ and HfAlO_X_ MOS-HEMT with the N_2_ plasma treatment.

The typical drain current-voltage characteristics of the Γ-gate HEMT-, HfO_2_-, HfAlO_X_-based MOS-HEMT, and non-recessed HEMT is shown in Figure 4. The maximum drain currents (I_DMAX_) of the HfAlO_X-_ (@V_G_ = 5 V) and HfO_2-_ (@V_G_ = 4 V) based MOS-HEMTs were found to be 870 and 775 mA/mm, respectively. Whereas, I_DMAX_ values (@V_G_ = 1 V) were found to be 570 and 520 mA/mm for the recessed and non-recessed HEMT, respectively, which is comparatively much lower than Γ-gate MOS-HEMT. Owing to the large gate leakage current, the HEMTs were not biased with the high gate voltage [32]. Henceforth, due to the insertion of the gate dielectrics the gate leakage current can be effectively suppressed, which resulted in the improvement of the I_DMAX_ in the MOS-HEMT. 

**Figure 5:**
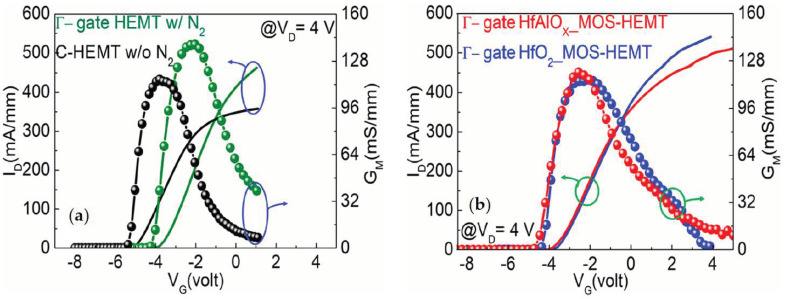
Comparison of transfer characteristics (I_D_–V_G_) (@ V_D_ = 4 V) of (**a**) Γ-gate HEMT (with the N_2_ plasma treatment) and non-recessed HEMT (w/o the N_2_ plasma treatment) and (**b**) Γ-gate HfO_2_ and HfAlO_X_ MOS-HEMT with the N_2_ plasma treatment.

To understand the gate controllability of the N_2_-plasma treated Γ-gate HEMT-, HfO_2_-, and HfAlO_X_-based MOS-HEMT and C-HEMT, the transfer characteristics were calibrated at V_D_ = 4 V, as shown in Figure 5. The threshold voltage (V_TH_) is defined as the gate bias intercept point of the linear extrapolation of I_D_ at peak transconductance (G_MMAX_) [33]. The threshold voltages were found to be −3.55, −3.41, and −3.55 V for Γ-gate HEMT-, HfO_2_-, and HfAlO_X_-based MOS-HEMT, respectively. In comparison, the V_TH_ of the non-recessed HEMT was found to be −4.9 V. The partially gate recessed and the use of field plate structure resulted in the positive shifting of V_TH_ in Γ-gate HEMT and MOS-HEMTs. The maximum transconductances were found to be 139, 116, and 118 mS/mm for the partially recessed HEMT-, HfO_2_-, and HfAlO_X_-based MOS-HEMT, respectively. The insertion of the gate dielectric layer, increased the distance between the gate and 2DEG channel. Therefore, the controllability of the gate is decreased which resulted in the reduction of the transconductance. The G_MMAX_ was found to be 114 mS/mm for the non-recessed HEMT.

**Figure 6:**
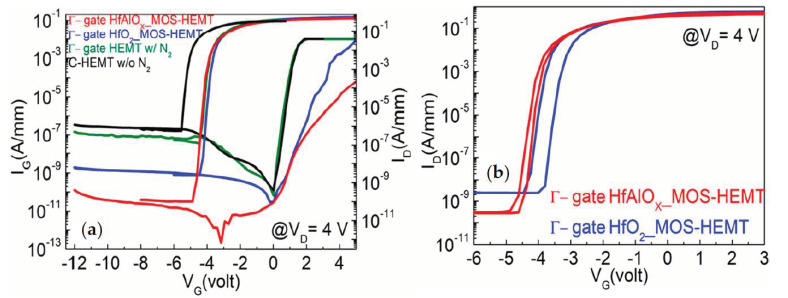
Comparison of (**a**) subthreshold (@ V_D_ = 4 V) and gate leakage (I_G_–V_G_) characteristics of Γ-gate HEMT and Γ-gate HfO_2_ and HfAlO_X_ MOS-HEMT with the N_2_ plasma treatment and non-recessed HEMT (w/o the N_2_ plasma treatment). (**b**) Hysteresis characteristics of (@V_D_ = 4 V) Γ-gate HfO_2_ and HfAlO_X_ MOS-HEMT with the N_2_ plasma treatment.

Figure 6a shows the subthreshold characteristics as a function of the gate voltage (@ V_D_ = 4 V) for Γ-gate HEMT-, HfO_2_-, and HfAlO_X_-based MOS-HEMT with the N_2_-plasma treatment, and non-recessed HEMT. From Figure 6a, it is clearly observed that the subthreshold drain leakage current was decreased more than two orders of magnitude in the HfAlO_X_-based MOS-HEMT, than the Γ-shaped gate HEMT. The HfAlO_X_ gate dielectric improved the metal/semiconductor junction which subsequently reduced the drain leakage current. The subthreshold drain leakage current is dominated by the reverse biased gate leakage current (I_G_) in the off-state [34]. Thus, the reduction of the reverse biased gate leakage current in the MOS-HEMT, resulted in the decrement of the subthreshold drain leakage current, as shown in Figure 6a. 

The subthreshold swing (SS) also depends on the I_G_. To understand the gate controllability, the SS values were extracted for different devices from Figure 6a. The SS values were found to be 101, 86, and 90 mV/dec for the N_2_-plasma treated partially recessed HEMT-, HfO_2_-, and HfAlO_X_-based MOS-HEMT, respectively. The current ON/OFF ratio (I_ON_/I_OFF_) of the aforementioned devices were found to be 2.9 $\times \;10^{6}$, 2.3 $\times \;10^{8}$, and 1.8 $\times \;10^{9}$, respectively. The SS and I_ON_/I_OFF_ were found to be 110 mV/dec and 1.04 $\times \;10^{6}$, respectively for the non-recessed HEMT. 

The reduction of the off-state gate leakage current is important in GaN HEMTs for their application in the electrical circuit. In general, it is also important to achieve a low flicker noise and low power consumption. The reversed and forward gate leakage I-V characteristics of the N_2_-plasma treated Γ-gate HEMT-, HfO_2_-, and HfAlO_X_-based MOS-HEMT and C-HEMT are shown in Figure 6a. It is clearly revealed that the reverse biased gate leakage current of Γ-gate MOS-HEMT was reduced more than two orders of magnitude than the Γ-gate or non-recessed HEMT due to the insertion of gate oxide as expected. The reversed biased gate leakage current (@ V_G_ = −12 V) were found to be $1.5\times 10^{-7}$, $1.7\times 10^{-9}$, and, $1.3\times 10^{-10}$ A/mm for the N_2_-plasma treated Γ-gate HEMT-, HfO_2_-, HfAlO_X_-based MOS-HEMT. In particular, the HfAlO_X_-based device exhibited the lowest I_G_ among all the devices. Due to the inclusion of Al in HfO_2_, the band gap increased from ~ 5.8 eV to greater than 6.5 eV for HfAlO_X_ with ~50% of Al, which is consistent with the large band gap of Al_2_O_3_ (~8.1 eV) [26]. The greater band offset of the HfAlO_X_ than HfO_2_, effectively reduced the tunneling current which results in the reduction of the gate leakage current [26]. It can also be noted that the forward leakage current (@ V_G_ = 5 V) also reduced more than three orders of magnitude in the HfAlO_X_-based MOS-HEMT than the other devices, due to the large band offset. In comparison with the other devices, for C-HEMT without FP and the N_2_ surface treatment, the I_G_ was found to be approximately (@ V_G_ = −12 V) $3.7\times 10^{-7}$ A/mm. 

To understand the hysteresis behavior of the gate dielectrics, the double sweep subthreshold transfer characteristics of Γ-gate HfO_2_ and HfAlO_X_ MOS-HEMT were measured, as shown in Figure 6b. It is clearly seen that the HfAlO_X_-based MOS-HEMT (~150 mV) exhibited a much smaller hysteresis than the HfO_2_-based devices (~484 mV). With the inclusion of Al into HfO_2_ the interface state density was reduced due to the effective passivation of the dangling bonds in HfAlO_X_ than HfO_2_ [30]. The incorporation of Al into HfO_2_ also reduced the border traps which resulted in the reduction of hysteresis in HfAlO_X_ [26]. 

**Figure 7:**
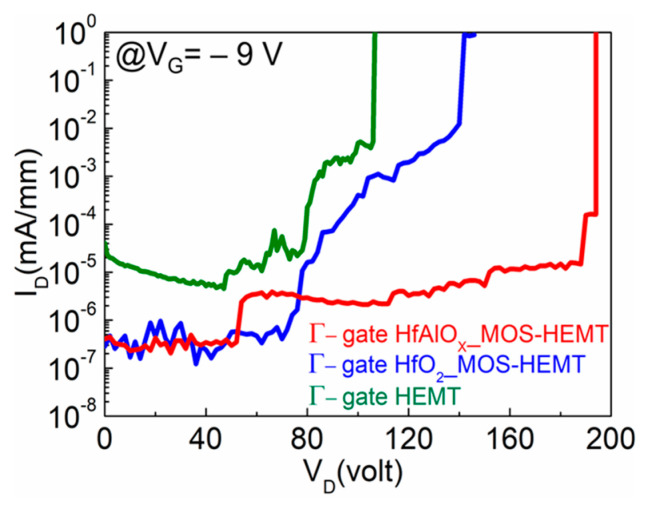
Comparison of breakdown voltage characteristics of Γ-gate HEMT, HfO_2_, and HfAlO_X_ MOS-HEMT with the N_2_ plasma treatment.

The off-state breakdown characteristics of different devices is shown in Figure 7. The breakdown voltages were found to be 107, 145, and 195 V for the Γ-gate HEMT-, HfO_2_-, and HfAlO_X_-based MOS-HEMT, respectively. The defect states caused by ICP dry etching increased the gate leakage, resulting in the significant reduction of the breakdown voltage (V_BR_) in the Γ-gate HEMT. As discussed earlier, due to the inclusion of Al into HfO_2_ the Γ-gate/HfAlO_X_ interface was improved with a greater dangling bond passivation with the increased band gap and reduced I_G_, which resulted in a much higher V_BR_ in the HfAlO_X_- based MOS-HEMT compared to the HfO_2_-based device. 

**Figure 8:**
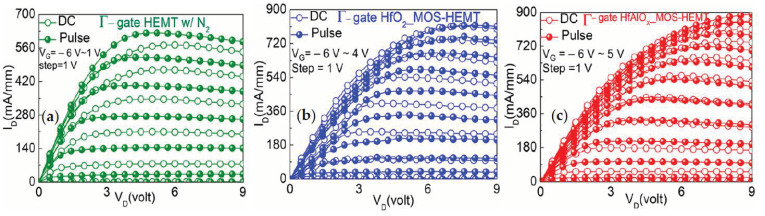
Comparison of pulsed I_D_-V_D_ characteristics of (**a**) Γ-shaped gate HEMT, (**b**) HfO_2_, and (**c**) HfAlO_X_ MOS-HEMT with the N_2_ plasma treatment.

The current collapse is an important issue for the electrical performance of AlGaN/GaN HEMTs, due to the electron trapping at the AlGaN surface states between the gate and the drain the current collapse occurred [31]. To investigate the effectiveness of the HfAlO_X_ gate dielectric with the field plate structure on the current degradation, the gate lag measurements were employed. Figure 8a–c shows the drain current response of the Γ-gate HEMT-, HfO_2_-, and HfAlO_X_-based MOS-HEMT with the field plate structure. The pulse width is 500 μs and the pulse period is 50 ms. From the observations, it was clearly revealed that the current collapse phenomenon was effectively suppressed with the gate dielectric in the partially recessed MOS-HEMT. The drain-source current collapse was improved in the HfAlO_X_-based MOS-HEMT to 4% (@V_D_ = 8 V, V_G_ = 0 V), while for the HfO_2_-based MOS-HEMT and partially-recessed HEMT it was found to be approximately 13% and 18%, respectively. Most of the surface states presented at the source-drain access regions might have been passivated with the combined effects of the N_2_ plasma treatment and HfAlO_X_ surface passivation with the application of the gate field plate.

**Figure 9:**
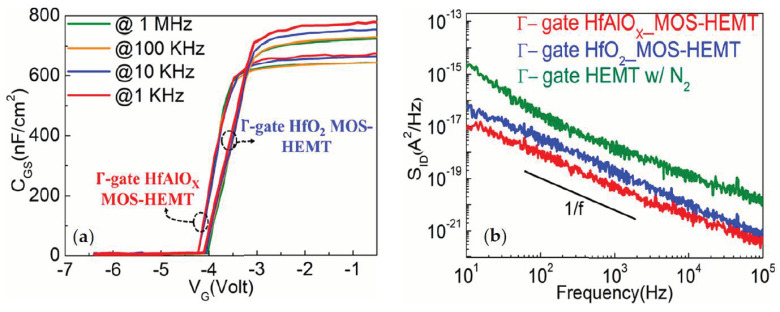
Comparison of (**a**) C-V characteristics of Γ-gate HfO_2_ and HfAlO_X_ MOS-HEMT at different frequencies and (**b**) flicker noise characteristics of Γ-gate HEMT, HfO_2_, and HfAlO_X_ MOS-HEMT with the N_2_ plasma treatment.

In order to understand the reduction of trap states of Γ- gate HfO_2_ and HfAlO_X_-based MOS-HEMTs, the capacitance-voltage (C-V) measurements were done for the devices at different frequencies, as shown in Figure 9a. The interface state densities (D_it_) were extracted from the previously reported formula [35] to be $1.8\times 10^{12}$ and 7.1 $\times \;10^{12}\;{\mathrm{eV}}^{-1}\cdot {\mathrm{cm}}^{-2}$ for the HfAlO_X_- and HfO_2_-based MOS-HEMTs, respectively. In addition, the interface quality between the gate dielectric and the GaN layer could also be evaluated from the frequency dependent C-V measurement shown in Figure 9a. The interval of the C-V curve improved to 60 mV from 130 mV for the HfAlO_X_ device, indicating the excellent interface quality between the gate dielectric and GaN layer [36,37]. 

Low-frequency noise measurements are an effective method for studying the electron trapping and de-trapping behaviour. Figure 9b shows the low-frequency noise characteristics, measured at V_DS_ = 2.9 V and V_GS_ = −3.9 V, with frequencies ranging from 10 to 100 kHz, for the Γ-gate HEMT-, HfO_2_-, and HfAlO_X_-based MOS-HEMT. In the noise characteristics, the variation of noise current density S_ID_ (A^2^/Hz) with the frequency was measured. The 1/f-noise characteristics are directly related to the presence of the electron trap and de-trapping between the 2DEG channels and the traps presented in the GaN buffer layer [38]. It was observed that S_ID_ of the Γ-gate MOS-HEMT was more than one order lower than the partially recessed HEMT. The gate dielectrics significantly reduced the gate leakage currents and passivated the S/D access region, which subsequently improved the interface quality, as discussed earlier. Consequently, the insertion of gate dielectric layer can suppress the flicker noise. In particular, due to the better interface quality with the lower interface density, the noise current density was found one order lower in HfAlO_X_-based MOS-HEMT (~10^−17^ A^2^/Hz) than the HfO_2_-based device (10^−16^ A^2^/Hz) at a low frequency. Table 1 shows the comparison of the N_2_ plasma treated Γ- gate HEMT-, HfO_2_-, HfAlO_X_-based MOS-HEMT and non-recessed HEMT. Table 2 shows the comparison of electrical performances of Γ-gate HfAlO_X_-based MOS-HEMT with different Al_2_O_3_ and HfO_2_-based MOS-HEMTs. The performance is comparable with the previous reports. 

## 4. Conclusions

In summary, we have demonstrated the comparative study of N_2_ plasma treated Γ- shaped gate AlGaN/AlN/GaN MOS-HEMT with HfO_2_ and HfAlO_X_ as gate dielectrics. The off-state gate leakage current was significantly reduced with the increasing PDA temperature from 300 to 600 °C in the HfAlO_X_-based MOS-HEMT compared to the HfO_2_-based devices. Due to the inclusion of Al into HfO_2_, the crystallization temperature was significantly improved to ~1000 °C of HfAlO_X_. Moreover, due to the higher conduction band offset of HfAlO_X_ to GaN (~1.61 eV), the gate leakage current was effectively reduced in HfAlO_X_ MOS-HEMT even at the higher temperature. The interface state density (D_it_) in the HfAlO_X_-based MOS-HEMT was effectively reduced compared to the HfO_2_ device, due to the effective passivation of the dangling bonds, which subsequently improved the hysteresis and current collapse characteristics. Due to the reduction of N_2_ vacancies, created by ICP dry etching, the gate leakage current was reduced more than one order of magnitude by the N_2_ surface plasma treatment. The D_it_ values were improved to $1.8\times 10^{12}$ ${\mathrm{eV}}^{-1}\cdot {\mathrm{cm}}^{-2}$ from $7.1\times 10^{12}\;{\mathrm{eV}}^{-1}\cdot {\mathrm{cm}}^{-2}$ for HfAlO_X_ MOS-HEMT than HfO_2_ MOS-HEMT. The N_2_ plasma treated Γ-shaped gate HfAlO_X_ MOS-HEMT exhibited a decent performance with I_DMAX_ of 870 mA/mm, G_MMAX_ of 118 mS/mm, threshold voltage (V_TH_) of −3.55 V, higher I_ON_/I_OFF_ ratio of approximately 1.8 $\times 10^{9}$, subthreshold slope (SS) of 90 mV/dec, a V_BR_ of 195 V with reduced current collapse of 4%, and gate leakage current of $1.3\times 10^{-10}$ A/mm.

## Figures and Tables

**Table 1:** Comparison of N_2_ plasma treated Γ-gate HEMT and MOS-HEMTs and C-HEMT w/o the N_2_ plasma treatment.

Parameters	Non-Recessed HEMT	Partially-Recessed HEMT	Partially-Recessed HfO_2_ MOS-HEMT	Partially-Recessed HfAlO_X_ MOS-HEMT
I_DMAX_ (mA/mm)	520 (V_G_ = 1 V)	570 (V_G_ = 1 V)	775 (V_G_ = 4 V)	870 (V_G_ = 5 V)
V_TH_ (V)	−4.9	−3.55	−3.41	−3.55
G_MMAX_ (mS/mm) (@ V_D_ = 4 V)	114	139	116	118
SS (mV/dec)	110	101	86	90
I_ON_/I_OFF_	1.04 $\times 10^{6}$	2.9 $\times 10^{6}$	2.3 $\times 10^{8}$	1.8 $\times 10^{9}$
I_G_ (A/mm) (@V_G_ =−12 V)	$3.7\times 10^{-7}$	$1.5\times 10^{-7}$	$1.7\times 10^{-9}$	$1.3\times 10^{-10}$
Current collapse (%)	-	18	13	4
D_it_ (${\mathrm{eV}}^{-1}\cdot {\mathrm{cm}}^{-2}$)	-	$2.1\times 10^{13}$	$7.1\times 10^{12}$	$1.8\times 10^{12}$
Hysteresis (∆V) (V)	-	-	0.48	0.15
V_BR_ (V)	-	107	145	195

**Table 2:** Comparison of Electrical performances of Γ-gate HfAlO_X_-based MOS-HEMT with Al_2_O_3_ and HfO_2_ MOS-HEMT.

Parameters	Ref. [21]	Ref. [23]	Ref. [39]	Ref. [40]	Ref. [41]	This Work
Epi structure	GaN/AlGaN/GaN	GaN/AlGaN/AlN/GaN	AlInN/AlN/GaN	GaN/AlGaN/GaN	GaN/AlGaN/AlN/GaN	GaN/AlGaN/AlN/GaN
L_G_ (µm)	1	5	0.2	50	1.5	0.5
Dielectric materials	HfO_2_/Y_2_O_3_	HfZrO_X_	Al_2_O_3_	Al_2_O_3_	Al_2_O_3_	HfAlO_X_
Dielectric Thickness (nm)	12/1	20	5	50	20	10
I_DMAX_ (mA/mm)	600	705	1150	~160	300	870
G_MMAX_ (mS/mm)	4.5 (@V_D_ = 0.05 V)	54 (@V_D_ = 10 V)	185	77 (@V_D_ = 15 V)	79 (@V_D_ = 8 V)	118 (@V_D_ = 4 V)
SS (mV/dec)	70	85	-	-	74	90
I_ON_/I_OFF_	10^9^	10^6^	-	-	$9\times 10^{8}$	1.8 $\times 10^{9}$
I_G_	10^−10^ A/mm (@V_G_ = −9 V)	~10^−7^ A/mm (@V_G_ = −10 V)	~10^−4^ A/mm (@ V_G_ = −15 V)	> 10^−5^ A/m^2^ (@ V_G_ = −5 V)	10^−11^ A/mm (@ V_G_ = -6 V)	$1.3\times 10^{-10}$ A/mm (@V_G_ = −12 V)
Current collapse (%)	-	< 9%	-	-	-	~4%
D_it_ (cm^−2^·eV^−1^)	10^12^	1.1 × 10^9^	-	-	-	1.8 × 10^12^

## Data Availability

The data presented in this study are available on request from the corresponding author.
